# Getting on “the same page”: a qualitative study on strategies for healthcare professionals in cross-cultural communication about serious neurological illness

**DOI:** 10.1186/s12904-025-01917-w

**Published:** 2025-10-27

**Authors:** Adela Wu, Karleen F. Giannitrapani, Gabriela D. Ruiz Colón, Karl A. Lorenz

**Affiliations:** 1 Department of Neurosurgery, Stanford University School of Medicine, 300 Pasteur Drive, Stanford, CA 94304 USA; 2 VA Health Services Research and Development Center for Innovation to Implementation, VA Palo Alto Health Care System, Department of Veterans Affairs, Palo Alto, CA 94304 USA; 3 Department of Primary Care and Population Health, Stanford University School of Medicine, Stanford, CA 94304 USA

**Keywords:** Serious illness, Goals of care, Communication, Strategies, Neurological illness, Facilitators

## Abstract

**Background:**

Serious illness communication with racial and cultural discordance can be challenging for both patients and providers. Furthermore, patients with serious neurologic conditions may have deficits that affect communication and medical decision-making. We aimed to explore strategies for effective culturally-tailored communication and interpersonal relationship-building that multidisciplinary healthcare providers use to facilitate serious illness conversations with patients of diverse cultural backgrounds.

**Methods:**

Using non-stratified purposive and snowball sampling, we conducted semi-structured interviews with 17 multidisciplinary providers, recruited from an academic tertiary care center, a Veterans Affairs hospital, and an academia-affiliated county hospital, who provide care for patients with serious neurologic conditions. We used inductive and deductive content analysis methods with dual review. We used inductive and deductive content analysis methods with dual review.

**Results:**

We describe six strategies providers used to establish trust and rapport with diverse patients and families. Theme 1: Recognize and check personal biases about race and culture. Theme 2: Create sufficient time and space to build connection with patients and families. Theme 3: Ask direct, open-ended questions to gather clear information about lived experiences and establish rapport. Theme 4: Repeat information as necessary to ensure understanding and bi-directional engagement. Theme 5: Understand family structure and dynamics and involve family members in communication and decision-making. Theme 6: Partner with palliative care teams, interpreters and other hospital staff as well as individuals from outside the healthcare system.

**Conclusions:**

High-quality care involves culturally-sensitive and tailored communication, which can be challenging for patients with neurocognitive deficits and limited abilities in communication and decision-making. Our study highlights six ways on how to establish rapport and improve cross-cultural serious illness communication with these diverse patients and families.

**Supplementary Information:**

The online version contains supplementary material available at 10.1186/s12904-025-01917-w.

## Introduction

The United States population reflects increasing diversity and growth in all racial and ethnic minority groups, according to the 2020 census [1]. Unfortunately, diversity in the healthcare workforce has not kept pace [2]. For example, the numbers of medical school matriculants of minority backgrounds do not reflect the proportions of black and Hispanic people in the general United States population [2]. While it is known that racial concordance between patients and their providers influences healthcare received, including patient satisfaction and communication, there continue to be many opportunities to improve engagement with patients and families in culturally-discordant contexts [3, 4, 5].

One such context is conversations about serious illness, which require aptitude with communication and interpersonal skills as well as cultural sensitivity. Patients diagnosed with serious neurological conditions, such as brain cancer, stroke, or traumatic brain injury, may also have impaired cognition or altered consciousness, further impacting providers’ facility and comfort with conducting serious illness conversations about goals of care (GOC).

Patients’ racial and cultural backgrounds impact healthcare delivery and access, and cultural respect may be integral in reducing health disparities [6]. Race and ethnicity are associated with readiness for serious illness care when patients perceived discrimination and grappled with mistrust and poor communication with healthcare providers [7]. Patients’ level of spirituality was also associated with quality of patient-provider communication, particularly with discerning religious beliefs and with involving patients in discussions about treatment preferences if the patient became unable to communicate [8]. Thus, it is imperative to understand areas for improvement in effective serious neurological illness communication, when providers must navigate difficult topics in a culturally-attuned manner.

In this qualitative study, we aim to explore various strategies for effective culturally-tailored communication and interpersonal relationship-building that multidisciplinary healthcare providers use to facilitate serious illness conversations with patients of diverse cultural backgrounds.

## Methods

### Study design and data collection

Semi-structured interviews were conducted by a MD-trained neurosurgery resident educated in qualitative interviewing methods (A.W.) and trained by a PhD-trained research team member with qualitative methods background (K.G.). The interview guide was developed and reviewed by a multidisciplinary research team with clinical (neurosurgery, palliative care) and methodological expertise (A.W., K.G., K.L.) (Supplementary File 1). All participation was voluntary and conducted in English. All identifying information was removed from all interview transcripts, which were numbered for reference. All study protocols were approved by the Veterans Affairs (VA) and Stanford Institutional Review Boards (IRB). Verbal consent to participate and audio-record all interviews was obtained at the beginning of each session.

The interview guide was created with principles from Levesque et al.’s conceptual framework of access to healthcare. [9] The interview guide covered opportunities and suggestions for strategies to improve cultural competency in discussing decisions with seriously ill patients and their families. The interview guide was pilot-tested in and further developed from two interviews with the same research aims as the main study.

### Participants

We interviewed healthcare professionals who cared for patients with serious neurosurgical and neurologic illnesses, including brain cancer, stroke, and traumatic brain injury, between December 2021 and June 2022. Table 1 includes demographic information about the study participants. In addition to possessing the aforementioned relevant clinical background and experience, eligibility criteria included English proficiency. Out of 19 providers approached for this study, two healthcare professionals declined to participate in interviews due to lack of time and availability for scheduling. Interviewed participants included neurosurgeons, inpatient advanced practice providers (APPs), neurologists, radiation oncologists, and social workers who are employed at any of the study sites (Stanford, Santa Clara Valley Medical Center, VA Palo Alto). Interviews occurred over private Zoom conferences with one interviewer (A.W.) from the research group. Each interview lasted between 20 and 45 min (average length: 35 min). Audio recordings were transcribed through an artificial intelligence-powered encrypted service and stored on a single, encrypted, HIPAA-complaint computer, and no transcripts were shared with participants.

**Table 1:** Characteristics of clinical provider participants Participant characteristics

Characteristic	N	%
Provider Type		
* MD*	*12*	*70.6%*
Neurosurgery	7	58.3%
Neurology	4	33.3%
Radiation Oncology	1	8.3%
* NP/PA*	*3*	*17.6%*
Neurosurgery	3	100%
Neurology	0	0%
Radiation Oncology	0	0%
* Social Worker*	*2*	*11.8%*
Neurosurgery	1	50%
Neurology	0	0%
Radiation Oncology	1	50%
Provider Workplace		
Stanford	12	70.6%
Santa Clara Valley Medical Center	2	11.8%
VA Palo Alto	3	17.6%
Gender		
Female	10	58.8%
Male	7	41.2%
Race/Ethnicity		
White	8	47.0%
Black	3	17.6%
Asian	5	29.4%
Multi-racial	1	5.9%
Hispanic	3	17.6%
Non-Hispanic	14	82.4%

Participants were recruited based on the interviewer’s prior knowledge of their healthcare experience and background and through snowball sampling. Non-stratified purposive sampling method was also used to create a group of interviewees with a range of specialty backgrounds [10]. There was no predetermined target number, and participants were recruited until thematic and code saturation were achieved from iterative data analysis and discussions of codes and themes by all members of the research team [11].

### Data analysis

The evaluation team consisted of one neurosurgeon (A.W.), one palliative care physician (K.L.), one PhD-trained qualitative methodologist (K.G.), and an analyst (G.R.C). Our two-part analysis leverages a combination of inductive and deductive approaches [12]. First, we summarized interviews on the day of completion with structured templates [13]. We used the information from the structured templates to draft an initial code list with definitions. Additionally, we (A.W. and G.R.C.) open-coded three transcripts to identify emergent concepts [14]. We then iteratively and collaboratively developed a codebook with definitions and finalized the codebook by group consensus [15]. Two researchers coded an additional two transcripts with the final codebook and compared and reconciled discrepancies in coding. Subsequently, all transcripts were coded by A.W., with code review by other investigators (Supplementary File 2). All codes were applied using Dedoose (Version 9.0.17), a qualitative analytic software. Research team members met bi-weekly for a period of six months to reconcile discrepancies in coding by consensus. We analyzed the excerpts tagged with “cultural identity”, which was assigned to any excerpt that specified the cultural background of patients and families, and “facilitators”, which referred to any mention of strategies or communication styles used by providers in serious illness communication. This combination of codes was assigned to 63 excerpts and subject to secondary analysis as we inductively developed and described emergent themes from these excerpts. Our study meets COREQ requirements for qualitative research [16].

## Results

Semi-structured interviews were conducted with 17 healthcare providers involved in caring for patients diagnosed with serious neurosurgical and neurologic disorders. Participants included 12 physicians (MD) from neurosurgery, neurology or radiation oncology specialties; three APPs (NP/PA); and two social workers (Table 1).

Thematic analysis elicited six themes regarding facilitators and strategies for improving cross-cultural serious illness communication and cultural sensitivity in providing care for diverse patients and families. Table 2 includes additional exemplary quotations from participants.

**Table 2:** Illustrative Excerpts. Abbreviations: advanced practice provider (APP)

Theme	Participant Role	Exemplary Quote
Theme 1: Recognize and check personal biases about race and culture.	Neurosurgery MD	“I think getting a sense of where they’re at and trying to accept any culturally diverse differences…things that are culturally very important and I don’t think it’s going to hurt them…I encourage them and try to embrace it and try to learn from it.”
	Social Worker	“When there are cultural considerations… what’s important to them and how they want to receive that information…How do we make sure to do that both culturally and ethnically when I’m setting up those discussions that will eventually be had with a team? Ideally, that’s what I like to do.”
	Neurology MD	“But, everyone comes to the table with their own biases and experiences and backgrounds. And so trying to figure out what’s important to people and meet them where they are helps.”
Theme 2: Create sufficient time and space to build connection with patients and families.	Neurosurgery MD	“The other thing that I usually do is, after three days, to call them. Just saying hi to see if everything is okay. I am not necessarily asking about making decisions. I’m not pressing them. The whole idea is to give them the feeling that there is somebody caring about them. So, I think it’s important to build the relationship. It makes everything much easier.”
	Neurosurgery APP	“In the past, if there is someone with some strong belief, I try and spend time to bridge that myself with the patient.”
	Neurosurgery MD	“They appreciate that you are giving them the time. They feel that you become part of the family. They know that I have a clinic, but I am dedicating my time to them, and they really appreciate it. You need to communicate to them you care about them. They understood the gravity of the situation.”
Theme 3: Ask direct, open-ended questions to gather clear information about lived experiences and establish rapport.	Neurology MD	“I try to ask open ended questions about what factors are important to them. I think it is important also not to make assumptions.”
	Social Worker	“I try to ask open questions when I’m doing assessments, like: “is there any religious-based preference or cultural things that are important to you that we should know about what we’re taking care of you?”
Theme 4: Repeat information as necessary to ensure understanding and bi-directional engagement.	Neurosurgery MD	“I have patience. Some patients and their families take longer than others to understand things and process it. You may have to repeat it multiple times… and being aware and sensitive and empathetic that people and families are different.”
	Neurology MD	“The first broad generalization that I have learned over time is to, first and foremost, expect that it’s going to take multiple meetings. To go into a family meeting, I know that people need to hear things many, many times or it’s not going to register.”
Theme 5: Understand family structure and dynamics and involve family members in communication and decision-making.	Neurosurgery MD	“I talked a lot with her daughter…almost using her as a proxy for all the detailed conversations that I would’ve had with the patient. I’m not sure whether that was the right thing but I felt that I needed to address and inform the family even more since I couldn’t do so with the patient.”
	Neurosurgery APP	“I feel like some families select one person to be in charge, and that person is consistent. And they’re usually the one person that shows and then they disseminate information to the rest of the group. But I would say I think that the Hispanic family structure just a little bit different. I send the information to the husband in this patient’s case and the son, but then the daughter is also very involved. So they were all kind of helping share information with the group.”
Theme 6: Partner with palliative care teams, interpreters and other hospital staff as well as individuals from outside the healthcare system.	Neurology MD	“In some cases where we make a recommendation, it’s very clear what the patient’s wishes are, but no decision was going to be made until their priest actually came in and joined the meeting. Then, we’re able to move forward.”
	Neurology MD	“It’s helpful when you have people from different backgrounds interact. And then something we do in our clinic, we go over each patient each week and discuss their case and some of the cases…the most active issue is a social or cultural issue. We discuss as a team and discuss approaches and ask each other for help. So I think that type of outlet and place where you can learn from other people, is very helpful.”
	Neurosurgery MD	“What we do have are lots of caring individuals from diverse backgrounds who, depending on what you need, may be there to help steer the conversation in a way that needs to be steered. Those include people in pastoral care. I’ve found them very helpful with people with certain beliefs, and they just want to make sure that their religious beliefs are cared for.”

### Theme 1: recognize and check personal biases about race and culture

Culture and patients’ personal lived experiences broadly influence their conception of and attitudes towards medical care:

“*whether [of] racial minority or majority*,* their experiences impact their decision making…and not every physician they see is going to align or have concordance in their racial background*” (Interview 9).

 Especially in racially-discordant patient-provider interactions,

“*rapport and good understanding are important when every patient brings their own personal story*,* personal beliefs about medicine*” (Interview 8).

Throughout the interviews, multiple healthcare providers also understood the limitations of their personal world views during interactions with diverse patients and families:

“*you can’t walk in anybody’s shoes*,* because we all come from different backgrounds. You can’t understand the magnitudes to which people go to*,* especially when you are from different cultures*” (Interview 17).

One provider finds it important to respect cultural differences and different values patients may hold,

“*because we just see a very small piece of who they are through the medical lens*,* but they’re diverse in other parts of their lives*” (Interview 4).

When queried about strategies to improve care delivered to diverse patients and families, healthcare professionals acknowledged that cultural sensitivity requires a mindful foundational awareness of patients’ unique backgrounds, a sentiment that arose throughout the majority of interviews:

“*if acknowledging that people come to their healthcare from different perspectives*,* it’s forced to be in your mind*,* and you’re more likely to recognize when there’s a discrepancy in what you and the patient are thinking and feeling*” (Interview 6).

 Healthcare providers strived to

“*empower patients and families to ask questions of their providers*” (Interview 9) and “*meet them where they are*” (Interview 15).

### Theme 2: create sufficient time and space to build connection with patients and families

Sometimes, a patient’s religious preferences necessitate longer conversations with their physicians, such as Jehovah’s Witnesses patients and their beliefs about blood transfusions when they make decisions about surgery:

“*you may [need] a longer conversation…to proceed along a different route*,* like a non-surgical or a minimally invasive option. Yes*,* those visits do take longer*” (Interview 12).

GOC discussions can be particularly stressful, and for

“*these serious conversations*,* it’s important to have protected time. It’s respectful…and shows that it’s not just a brief update and can do a long way in establishing trust and rapport in helping patients make decisions*” (Interview 4).

Healthcare professionals are aware that their patients and families may struggle with serious illness communication, and one

“*booked [the family] for double the time*,* so they had time for ample questions*” (Interview 17).

Overall, interviewees express a desire to

“*spend time and be empathetic and willing to learn from the patient*” (Interview 17).

### Theme 3: ask direct, open-ended questions to gather clear information about lived experiences and establish rapport

Part of successful rapport-building hinges on understanding patients’ diverse lived experiences. To do so, several providers recognized that asking direct and open-ended questions to gather information was essential:

“*what ultimately changed [the conversation] was one of the palliative care attendings just asked a very open-ended question about what do they value in depth?*” (Interview 14).

Other examples of questions the interviewee suggested included

“*trying to see what are they worried about? What do they understand? What do they misunderstand? What is their fear? And try to address those head on*” (Interview 14).

Along with giving patients and families opportunities to express their views in response to open-ended questions,

“*not making assumptions is key…[by] confirming what I heard them trying to convey*” (Interview 9).

### Theme 4: repeat information as necessary to ensure understanding and bi-directional engagement

A challenging aspect of serious illness communication arises when patients and families may not be ready to process medical information, which a few interviewees directly addressed. Providers have the important role of facilitating and ensuring understanding and bi-directional engagement, particularly if there are language barriers, concerns with health literacy, or when families express mistrust in the American healthcare system: “*we had to spend a lot of time talking*,* …reiterating*,* and a lot of teachback to make sure that everyone understood what was happening and on the same page*” (Interview 14). Often, repetition of important information requires “*multiple meetings [because] people need time to process*” (Interview 15) or “*teaching aids or [involvement] of other people*,* like nurses*,* social workers*,* and other doctors*” (Interview 11) for effective communication.

### Theme 5: understand family structure and dynamics and involve family members in communication and decision-making

Family members are frequently involved in patient care, particularly in serious neurologic illness. Overall, interviewed providers broadly upheld family engagement in serious illness communication, finding it valuable to

“*let the family bring in as many voices as they can…and just listen. …It gives clues [about] how to talk to someone about where things are at*” (Interview 8).

Challenges may arise from differences in family structure and dynamics, particularly as providers interact within a culturally-disparate context. In general, healthcare providers describe the importance of acknowledging that

“*even within a family*,* family members are different…just be aware of that and try to adjust*” (Interview 11).

In addition to differences in family dynamics, family structures and the individuals who desire to be involved in communication may also be unique to each case. As a result, healthcare professionals adapt when

“*this family’s structure is…different…I send information to the husband…and the son in person*,* but the daughter is also very involved on Zoom*” (Interview 1).

Ultimately, even despite logistical barriers to arrange family group meetings for serious illness communication,

“*having everyone*,* [some] that the patient trusts and is close with*,* on the same page…and in the same room for a meeting…is important*” (Interview 13).

### Theme 6: partner with palliative care teams, interpreters and other hospital staff as well as individuals from outside the healthcare system

Serious illness communication benefits from teamwork and collaboration both within and external to the hospital setting. First, participants recognized the varied expertise and clinical knowledge different specialty teams brought to goals of care discussions:

“*having other clinicians…medical or surgical ICU teams… supporting you to say ‘this is what’s going on respiratory wise*,* et cetera*” (Interview 2).

Multidisciplinary team engagement may also facilitate building trust as a neurosurgeon described negative attitudes some patients and families may have towards surgeons:

“*there are some people who don’t believe neurosurgeons’ talk about recovery after neurologic injury*,* and they need someone who is not the surgeon*,* [such as] neurocritical care*,* to explain*” (Interview 8).

Uniformly, participants appreciate the invaluable services provided by certified medical interpreters when interacting with families that do not speak English as a primary language:

“*having somebody able to speak in the primary language of the patient and family…to clarify challenging points…is very helpful*” (Interview 1).

In some cases, medical translators may also serve as

“*cultural brokers*” (Interview 4).

Many interviewed providers also expressed interest in inviting important community members, such as

“*a specific member from their own church*” (Interview 14) or “*spiritual leaders in the community…whether or not it’s religious leaders*” (Interview 15).

The general consensus from healthcare providers highlighted the positive effect of discussing difficult conversations as a team:

“*that outlet…where you can learn from other people is very helpful*” (Interview 10).

## Discussion

Our analysis derived six strategies used by multidisciplinary healthcare providers to facilitate serious neurologic illness communication and interpersonal relationship building with diverse patients and families. Effective communication about serious illness and providing culturally-competent care are important opportunities to improve care for people from minority groups and educate healthcare providers. While it is possible to increase GOC discussion and documentation through interventions that prompt and guide GOC conversations, these randomized trials did not necessarily focus on patients from racial minorities [17, 18]. In our study, interviewees who provide care for seriously ill neurosurgical patients shared several strategies that they used to check personal biases as individuals in encounters with patients from different racial, religious, and cultural backgrounds.

People from racial minority backgrounds often experience disparities in end-of-life and serious illness communication. Hispanic, Asian, and Black patients are significantly less likely to have GOC discussions with their healthcare providers and document their treatment preferences, thereby impacting healthcare received near end of life [19, 20]. Based on results from focus groups with white, African American, and Latino participants, promoting clear and culturally-appropriate serious illness communication and recruiting providers who share common background with their patients were frequent suggestions to improve end-of-life care for diverse populations [21].

Cultural sensitivity training in the medical field has persisted in part due to institutional efforts promoting diversity, equity, and inclusion. This education takes many forms, including simulation modules and panels reflecting patient voices [22, 23]. Fewer studies evaluate the impact of cultural competence training on patient satisfaction reported by members of minority groups. A systematic review of seven studies found that six interventions significantly increased providers’ cultural competency and five studies established that these provider-oriented trainings improved patient satisfaction [24]. Our study delves into opportunities providers may heed to communicate and establish rapport in a culturally-attuned manner despite cultural discordance.

Some strategies outlined in our study are applicable for all patients and are reflected in prior literature, such as the Serious Illness Conversation Guide, which includes questions to guide and support clinicians with difficult conversations [25]. Interviewed providers identified these common facets of effective communication, including asking open-ended questions and ensuring information exchange over time through repetition.

When recounting interactions with diverse patients with different identities than their own, every healthcare provider mentioned the importance of checking personal implicit biases. Implicit biases are unconscious processes involving unintentional behaviors arising from personally held attitudes and stereotypes [26]. Prior literature has shown that providers’ implicit biases were negatively associated with not only length but also quality of patient communication in racially discordant provider-patient encounters [27]. However, few studies focus on steps taken to address implicit biases that contribute to health disparities, and these trainings typically consisted of online modules or case studies [28]. From the field of applied behavioral analysis, one review proposes several strategies to mitigate implicit biases towards race held by clinicians, from asking open-ended culturally-attuned questions to engaging caregiver input and paying attention to both vocal and nonvocal behaviors towards patients [26]. Several pilot studies about interventions addressing providers’ unconscious bias demonstrated increased self-reported awareness of race in patient care and increased knowledge, comfort and rapport with racial minority groups [29, 30].

Our study also highlights the importance of partnerships with families, other providers, and significant figures in patients’ lives outside of the medical setting for serious illness communication. Family members are involved in decision-making and GOC conversations, particularly when patients suffering from serious neurologic conditions may be nonverbal or unable to participate in discussions [31]. Providers also recognized the importance of cultural values, including religious beliefs, patients and their families held when facing serious illnesses. For example, African American families with higher levels of spirituality who reported higher quality of communication with providers experienced lower levels of conflicts with their decisions [32]. A group of providers involved in spiritual care and palliative care presented practical considerations for addressing patients’ religious and spiritual beliefs during serious illness [33]. They recommend that hospital chaplains can contribute to GOC conversations. Several clinicians in our study also involved important religious leaders from patients’ communities as support during serious illness discussions.

### Limitations

Our sample size is small (17), but we interviewed providers from diverse personal and professional backgrounds through purposive sampling [34]. All providers recounted work experiences in the San Francisco Bay Area, but we sought perspectives from a variety of practice settings and healthcare systems (academic tertiary referral center, county hospital, Veterans Affairs). Participants shared specific examples about their patients and families from various backgrounds, but we were not able to comment on data specific to a minority group due to insufficient sampling. Furthermore, trustworthiness in qualitative research consists of multiple aspects, including credibility, transferability, dependability, and confirmability. While our research team did not engage in certain practices, such as member checking or reflexivity, our team members were involved in peer debriefing as well as methods documentation and auditing [35].

## Conclusions

With growing diversity in the general patient population, healthcare providers recognize the importance of and need for cultural sensitivity, particularly in serious neurological illness communication. Our study highlights six strategies and practices providers personally employ to facilitate goals of care conversations and other difficult topics for patients and families from diverse backgrounds. Participating healthcare providers broadly expressed interest in longitudinal and dedicated cultural sensitivity training, and future work may investigate educational opportunities that also incorporate these communication and interpersonal skills strategies.

## Supplementary Information

Supplementary Material 1.

Supplementary Material 2.

## Data Availability

The data that support the findings of this study are not publicly available but can be available on reasonable request from the corresponding author, AW.
