# Supplementary material for: Getting on “the same page”: a qualitative study on strategies for healthcare professionals in cross-cultural communication about serious neurological illness
Source: BMC Palliat Care. 2025 Oct 27;24:271. doi: 10.1186/s12904-025-01917-w (PMC12560552; doi:10.1186/s12904-025-01917-w)
Supplement: Supplementary file 1 — Supplementary Material 1. [file 12904_2025_1917_MOESM1_ESM.docx]

Supplementary Table 1 - Providers Interview Guide

| **Questions** | **Second-Level Questions** |
| --- | --- |
| 1. Please describe your role and how long you have been in this role at this facility. |  |
| 1. I’d like to specifically talk about your seriously ill patients. What are some of the important decisions that you’ve discussed with patients and families? | What are additional important decisions you think that families and patients should consider? |
| 1. **What were some challenges you faced when communicating with patients about next steps in their course?** | How did you talk about your recommendations for the patient and family?  **How do you talk to a patient or their family when they don’t agree with your recommendations?** |
| **I’d like to now focus on “goals-of-care planning” – the discussion between a provider and the patient about their hopes, fears, and values.** Some people propose that with a serious illness, this should happen regularly. Can you discuss whether you think that’s a good idea or not? |  |
| If we were going to try to make these goals-of-care conversations more common, how would you recommend we go about that? | **Who should initiate or do the bulk of goals-of-care planning discussions with the patient?**  **How and when should palliative care specialists be involved?** |
| 1. **If you have done so before, what were some challenges you faced while addressing goals of care, particularly those with a different background from you?** | Did you have problems with:  -lack of time?  -comfort level with goals of care discussions?  -difficulty inserting language about goals of care?  Which part was especially challenging:  -interaction with family?  -patient health literacy?  -patient cultural values? |
| 1. I’d like us to think about a specific patient you’ve had.   Can you recall and share an example when that patient’s identity was important to their care? | Can you share examples of when their identity influenced decision-making and your conversation?  Was this patient’s background different or like yours? In what ways?  How did you navigate that interaction? |
| 1. What worked best when addressing challenges you had communicating with diverse patients, particularly those with a different background from you? | Do you ever bring up aspects of your patient’s identity during shared decision-making? |
| 1. What could we do to better prepare clinicians for addressing diverse cultures that we encounter when providing care? | What programs for improving cultural competency does your workplace offer? |
