# Supplementary material for: Getting on “the same page”: a qualitative study on strategies for healthcare professionals in cross-cultural communication about serious neurological illness
Source: BMC Palliat Care. 2025 Oct 27;24:271. doi: 10.1186/s12904-025-01917-w (PMC12560552; doi:10.1186/s12904-025-01917-w)
Supplement: Supplementary file 2 — Supplementary Material 2. [file 12904_2025_1917_MOESM2_ESM.docx]

Supplementary Table 2: Example quotes tagged with codes

| **Code** | **Interviewee Role** | **Exemplary Quote** |
| --- | --- | --- |
| Facilitator/Strategy | Neurosurgery APP | That [having more interpreters] would be really helpful because then you could just actually speak one to one. Families are there and they can help us interpret, but I think things get lost in translation. …Yeah, I think sometimes more time to explain is also helpful so that they would open up and feel more comfortable. |
| Facilitator/Strategy | Radiation Oncology Social Worker | We have some people with a lot of manic thoughts. They’re flooded. You have to tell them, “can you stop just a moment? I’m just noticing that you’re really revved up and just take a moment to pause and settle down.” I might even get them water. Just to orient them. |
| Cultural Identity | Neurosurgery MD | I have definitely seen, much more in Asians than others, especially older parents who don’t speak English. The kid wants to translate but they don’t actually want to tell the parents the diagnosis. That happens quite a bit. That’s to me a no-go. I tell the patients upfront their…it’s my moral obligation, to tell them the diagnosis. |
| Cultural Identity | Neurology MD | I may have one or two African American patients per year. Culturally, I would say the harder conversations there are the palliative care discussions. I think that as a society we’ve done so much damage to trust in physicians that even if you have a good relationship with those families, there seems to be a lack of trust. |
